# Key Intervention Categories to Provide Person-Centered Dementia Care: A Systematic Review of Person-Centered Interventions

**DOI:** 10.3233/JAD-210647

**Published:** 2021-10-26

**Authors:** Wiebke Mohr, Anika Rädke, Adel Afi, David Edvardsson, Franka Mühlichen, Moritz Platen, Martina Roes, Bernhard Michalowsky, Wolfgang Hoffmann

**Affiliations:** a German Center for Neurodegenerative Diseases e.V. (DZNE), Site Rostock/Greifswald, Greifswald, Germany; b Department of Nursing, Umeaa University, Umeaa, Sweden; c School of Nursing and Midwifery, La Trobe University, Melbourne, Australia; d German Center for Neurodegenerative Diseases e.V. (DZNE), Site Witten, Witten, Germany; e Institute for Community Medicine, University Medicine Greifswald (UMG), Greifswald, Germany

**Keywords:** Alzheimer’s disease, dementia, patient-centered care, patient-focused care, patient preferences, person-centered care, person-centered dementia care, person-centered interventions, psychosocial intervention

## Abstract

**Background:**

Person-centered care (PCC) is an important concept in many countries’ national guidelines and dementia plans. Key intervention categories, i.e., a taxonomy of person-centered (PC)-interventions, to provide person-centered dementia care, are difficult to identify from literature.

**Objective:**

This systematic review aimed to identify and categorize published PC-interventions into key intervention categories to guide the provision of person-centered dementia care.

**Methods:**

Conduct of this systematic review followed Cochrane guidelines. A search of the dimensions ‘Dementia’, ‘Person-Centered Care’, and ‘Intervention’ combined was performed in PubMed, EMBASE, and Web of Science. Study selection was based on 2-stage screening against eligibility criteria, limited to controlled study designs. Information about interventions and outcomes was extracted into an “Effects Table”. The identified PC-interventions were categorized in intervention categories to provide person-centered dementia care.

**Results:**

Searches identified 1,806 records. 19 studies were included. These covered a range of psychosocial interventions, oftentimes multi-component interventions, which followed heterogeneous approaches. Studies were conducted in long-term care/hospital settings. Nine key intervention categories were identified: social contact, physical activities, cognitive training, sensory enhancement, daily living assistance, life history oriented emotional support, training and support for professional caregivers, environmental adjustments, and care organization.

**Conclusion:**

Our findings provide a current overview of published PC-interventions in dementia, which followed heterogeneous approaches under the PCC-concept. The heterogeneity made it challenging to identify a well-defined concept of PCC and common key intervention categories. An effectiveness-evaluation of “PC” - including “relationship-centered”-interventions may be valuable, to assess whether an explicit focus on relationships around PCC-interventions yields an added benefit.

*PROSPERO-ID:* CRD42021225084.

## INTRODUCTION

With aging populations, dementia increasingly represents a challenge for public health and health care systems worldwide [1]. Globally, around 50 million people have dementia, and there are nearly 10 million new cases every year [2]. According to findings from the *Global Burden of Disease Study 2019*, Alzheimer’s disease and other dementias were the fourth leading cause of death globally in the age groups 75 years and older [3]. Despite the recent approval of aducanumab for Alzheimer’s disease by the U.S. Food and Drug Administration [4], no curative treatment for all people living with dementia (PlwD) exists. PlwD need a timely differential diagnosis and care, which ensures a high quality of life (QoL) [1, 5].

Person-centered care (PCC), a prominent concept in dementia care, has been suggested synonymous with good quality care [6]. Many countries include a PCC-approach in their national guidelines and dementia plans [7–13]. The concept is covered by a multiplicity of terms in the literature, dependent on the context in which care is provided. It challenges the traditional clinician-centered or disease-focused medical model to a model of care, which is customized to each person [14]. Some argue, PCC’s origins trace back to Florence Nightingale, “who differentiated nursing from medicine by its focus on the patient rather than the disease” (p. 246) [15]. Carl Rogers’ work on client centered psychotherapy noted “person-centeredness” in the early 1940s [16]. Until Tom Kitwood in 1988 noted PCC-approaches in dementia care [17], the term had not been used in the dementia care field [18]. Often, Kitwood is described as the founder of the concept of person-centered dementia care [19], developed in response to the reductionist regarded biomedical view of dementia, which downgrades the person to a carrier of a chronic disease and hereby ignores personal experiences, well-being, dignity, and worth [20, 21]. Despite the prominence and frequent use of PCC, some have noted the missing consensus or explicit agreement on its definition, the complexity of the concept, and a related need for more clarification [22–24]. Some have questioned, whether PCC is achievable [25], while others pointed out that PCC indirectly emphasizes autonomy and independence rather than the importance of relationships [26], even though Kitwood noted relationships as essential to understand dementia [19]. Relationship-centered care (RCC) may be seen as the next development of PCC, which pays more attention to the reciprocity of care between the care recipient and the caregiver (CG), by some [27, 28].

What PCC means in in clinical practice has been described broadly; it includes the incorporation of personal knowledge of the PlwD, to conduct meaningful activities, to make well-being a priority, and to improve the quality of relationships between the health care professional and the PlwD [18, 29]. Based on a non-pharmacological and sociopsychological treatment approach, PCC recognizes the need to personalize and tailor care to the recipient’s needs and preferences to guide care provision [30, 31]. Previous PCC-literature has focused on its theory and theoretical frameworks [18, 19, 30, 32–35], qualitative studies about the understanding of PCC [29, 36, 37], and tools to measure PCC [38–42]. Earlier published reviews of PCC for PlwD showed beneficial effects to manage challenging behaviors (such as agitation), reduce the use of antipsychotic drugs, neuropsychiatric symptoms, depression, and to improve QoL, as well as to improve healthcare professionals’ quality of work-life [43–47].

However, to the best of our knowledge, no previous review has tried to identify key intervention categories to guide the provision of person-centered dementia care, including who does what, where, and how, from the published literature. Hence, the following research questions arose:

1 What are the characteristics of published PCC-interventional studies for PlwD?
2 How can the interventions described in PCC-interventional studies for PlwD be synthesized into categories to guide the provision of person-centered dementia care?
3 What a) content, b) provider, c) format, d) setting, e) intensity, and f) fidelity describe key intervention categories to provide person-centered dementia care?

## METHODS

For the identification of key intervention categories, we performed a systematic review of PC-interventions for PlwD. The review was guided by the established guidelines in the *Cochrane Handbook for Systematic Reviews of Interventions*. For this report, the PRISMA Checklist was followed [49], which can be reviewed in Supplementary Table 1.

### Protocol and registration

A protocol for the review was registered with PROSPERO (Reference/ID No: CRD42021225084). We strictly followed this protocol for the systematic review process. For the report of our findings, we have adjusted some terminology for clarity and refocused the discussion and application of results to make the review suitable for a broader audience.

### Study eligibility criteria

The definition of eligibility criteria for this systematic review was based on the PICOS (Population, Intervention, Comparison, Outcome, Study Design) format of study design questions [50]. Records were included/excluded if they met the criteria as depicted in Table 1.

**Table 1:** Inclusion/exclusion criteria

Population	*Include:* Studies that include dementia populations as main group of study participants from any setting, who had any type of dementia diagnosed by health professionals. The dementia may be mild, moderate or severe.
	*Exclude:* Publications focused on non-human populations, persons with other diagnoses than dementia, or populations with mild cognitive impairment (MCI). Publications where the study a) investigates effects of interventions on or b) merely is tailored towards other persons than the People living with Dementia (PlwD) themselves, e.g., informal caregivers (CGs) or healthcare professionals.
Intervention	*Include:* Interventional studies, which focus on Person-Centered Care (PCC) applying the following terminology: a) “person-centered care” or respective synonyms as identified in the search string (see Supplementary Material 2) or b) highlight the perspectives, needs and preferences of the individuals studied.
	*Exclude:* Any studies that did not describe a health or social care interventional study. “Interventional study” is defined based on the WHO-definition for “health intervention”: “*A health intervention is an act performed for, with or on behalf of a person or population whose purpose is to assess, improve, maintain, promote or modify health, functioning or health conditions.*” [95]
Comparators	*Include:* Care as usual or placebo. For some groups, this may include pharmacological interventions.
	*Exclude:* Any publication that did not include a control group.
Outcomes	*Include:* At least one of the following outcomes for the PlwD had to be reported in the study:
	1. Time to care home admission/institutionalization
	2. Hospital admissions
	3. Quality of Life (QoL)
	4. Well-being
	5. Activities of daily living (ADLs)
	6. Behavior (e.g., neuropsychiatric symptoms, NPS)
	7. Cognition
	8. Mood (e.g., level of depression)
	9. Acceptance and adherence
	10. Satisfaction
	11. Social participation
	12. Overall survival (OS)
	13. Progression free survival (PFS)
	14. Use of medication
	15. Falls
	16. Hydration
	*Exclude:* Any publication that did not report any outcome measures. Any publication that did not report at least one of the patient-relevant outcomes for PlwD as listed above.
Study Design	*Include:* Only original research, concretely studies designed as Randomized Controlled Trials (RCTs) and Non-Randomized Controlled Studies (NRS) [96], e.g., non-randomized controlled trials, controlled before-and-after studies, interrupted time series studies, historically controlled studies, cohort studies, case-control studies and cross-sectional studies, which report patient relevant outcome measurements of PC-interventions, were included.
	*Exclude:* Any publication that was not available in English or German language. Publications not available as a full text journal article (i.e., conference abstracts or proceedings, books, letters or correspondence, editorials), or those that do not describe the methodology of investigation, were excluded. Similarly, reviews, protocols, pilot/exploratory studies, case reports, professional discussions, opinion pieces and descriptive studies of general service use not involving a designated intervention, as well as all qualitative research were excluded.

### Information sources and search strategy

The three dimensions, 1) Dementia, 2) Person-Centered Care, and 3) Intervention, were used for the development of the search strategy. The keywords used (see Supplementary Material 2 for complete search string) included Dementia (MeSH), Alzheimer’s Disease, Patient-Centered Care (MeSH), Person-Centered Care, Relationship-Centered Care, and all possible synonyms to this concept as identified via the MeSH-database [51] and previous literature, e.g., [38, 52, 53], in U.S.- and U.K.-English spelling, as well as Therapy (MeSH), intervention, and treatment, focused on those of non-pharmacological and psychosocial nature. The search was piloted prior to the development of the protocol. Time period restriction was not applied, language was limited to English and German. The databases searched included PubMed, Web of Science, and Embase, following recommendations from Bramer et al. [54] for optimal database combinations in literature searches. The last search was conducted on November 5, 2020.

### Study selection

De-duplication of identified records followed the systematic approach by Bramer et al. [55]. The first stage of study selection entailed the screening of titles and abstracts, performed by two reviewers (WM and AA). The screening process included to compare information presented in the title and abstract with the pre-defined in- and exclusion criteria. Eventual discrepancies were resolved by discussion between reviewers (WM and AA) until consensus was reached, and where this was not possible, a third researcher (AR, BM, FM, or MP) was consulted. All records where titles and abstracts were considered to conform with the eligibility criteria were included for full-text screening. The second stage of data selection, full-text review, performed by two reviewers (WM and AA), followed the aforementioned strategy. Both stages of the screening process were performed in the online software Rayyan [56]. Per PC-interventional study, only one published record in accordance with eligibility criteria was included.

### Data extraction

The following information was collected: author, country, setting, sample size, age in years of the target group, intervention, control group, duration/follow-up, dementia severity based on stated scores and/or stages, outcome measures, and study design. To organize the evidence data were entered into an “Effects Table”, a qualitative tool to display a concise summary of the included studies’ interventions and outcomes/effects.

### Risk of bias assessment

Two reviewers (WM and AA) examined the risk of bias for all included studies by application of two validated analysis tools: 1) the Cochrane Collaboration’s Risk of Bias tool 2 (RoB2) [57] for randomized controlled trials (RCTs) and 2) the Newcastle-Ottawa Scale (NOS) [58] for cohort-studies to assess the quality of the non-randomized controlled studies (NRS). Where discrepancies arose, a third researcher (AR, BM, MR, WH) was involved in the discussion.

### Data synthesis

A concise narrative summary was undertaken to identify key intervention categories. PC-interventions were analyzed for the distinct activities performed under their scheme, and respectively synthesized and categorized into named intervention categories with shared characteristics oriented in Dickson et al. [59], and Clarkson et al. [60]. The synthetization and categorization covered information about a) content (individual PC-interventions), b) provider(s), c) format, d) setting, e) intensity, and f) fidelity [61] for the distinct intervention categories.

## RESULTS

### Study selection

**Fig. 1:**
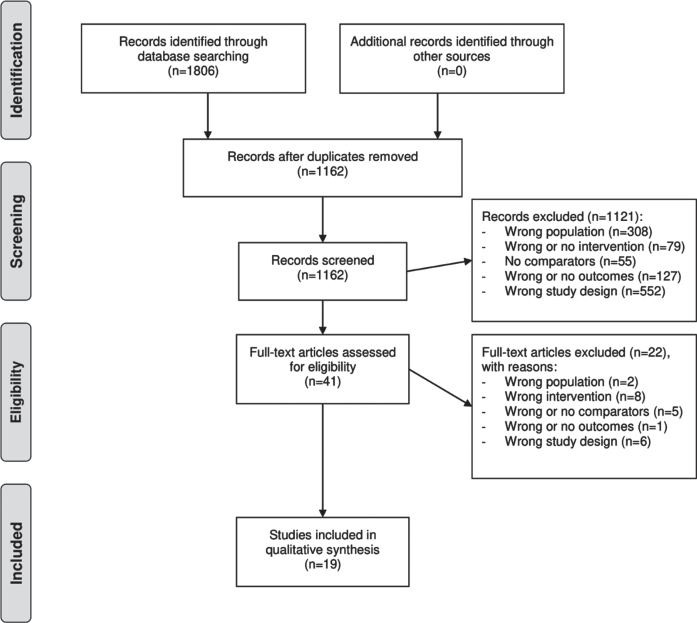
Study flow diagram. Note: Moher D, Liberati A, Tetzlaff J, Altman DG, The PRISMA Group (2009) Preferred Reporting Items for Systematic Reviews and Meta-Analyses: The PRISMA Statement. *PLoS Med* **6**, e1000097. For more information, visit http://www.prisma-statement.org.

The searches identified a total of 1,806 records. After removal of duplicates, 1,162 records were identified for title/abstract-screening, out of which 41 records underwent full-text review. The majority of records were excluded because of ineligible populations or study designs. The selection process is depicted in Fig. 1.

Following the screening of the full texts of selected records, 19 interventional studies were identified. 14 studies of those applied a RCT-design [62–75], and one study further used a quasi-experimental pre- and post-test design including randomization [76]. The remaining four [77–80] applied NRS-designs, including one cohort [78] and three non-randomized quasi-experimental, prospective, longitudinal studies [77, 79, 80].

### Characteristics of included studies

The summary of characteristics for the 19 included studies is depicted in Table 2. The summative Table 2 covers the information extracted and organized in the aforementioned Effects Table in a comprehensive display.

The majority of studies were conducted in high-income countries in Northern-America and Europe (USA [65, 68, 70, 75], UK [62, 67], Australia [63, 64, 74], Canada [66], the Netherlands [73, 76, 77, 79], Belgium [72], Norway [69, 71], Spain [80]) and in Asia (Singapore [78]). 18 studies were conducted in long-term care facilities without further specification on the operational model of the respective institutions (for profit or not for profit), one study was conducted at a hospital [78]. No studies were conducted in homecare / primary care settings. Sample sizes varied between 52 to 847. The majority of participants in the studies were, on average, above 80 years of age. The studies covered a wide range of interventions, oftentimes delivered as multi-component interventions [62–65, 67–69, 73, 74, 77–79]. Eight studies concretely stated an assessment of preferences or needs prior to the intervention [63–66, 72, 75–77], among which three [72, 75, 76] assessed preferences or needs by direct involvement of the PlwD, while the remaining relied on information from care plans and/or informal and professional CGs. Others mentioned the necessity to adjust the intervention to the PlwD’s preferences and needs, but did not report concrete assessments of the latter [68–71, 73, 74, 78]. Three studies [65, 69, 74] used placebo interventions, while the remaining provided usual care in the control group. The duration of the studies ranged from two weeks [65] up to 18 months [76]. Dementia severity varied, with many participants at moderate to severe stage. Seven studies found a significant positive effect on QoL [62, 63, 66, 77, 78, 80], nine studies found a significant positive effect on agitation [62–65, 70–72, 74, 78]. A comprehensive list of outcome measures including the respective measurement tools and an indication of effectiveness is depicted in the right column of Table 2.

**Table 2:** Narrative summary of characteristics for included studies

Author	Country	Setting	Sample size (N)	Age in years mean (SD)	Intervention	Control group	Duration/ follow-up	Dementia severity	Outcome measures^b^
**RCTs**
Ballard et al. [62]	UK	Nursing home	847	88.5 (0.50)	The **WHELD** program, which combined:	TAU	9 months	FAST stage:	*Primary:*
					1) staff training (training in PCC for staff and promoting tailored person-centered activities and social interactions), 2) social interaction, and 3) guidance on use of antipsychotic medications			*Mild or less*	- **QoL (DEMQOL-Proxy)**
								TAU: 35 (7.90%)	*Secondary:*
								WHELD: 47 (11.64%)	- **Agitation (CMAI)**
								*Moderate*	- **NPS (NPI-NH)**
								TAU: 38 (8.58%)	- Antipsychotic use (Med. charts)
								WHELD: 39 (9.65%)	- Global deterioration (CDR)
								*Moderately severe*	- Mood (CSDD)
								TAU: 267 (60.27%)	- Unmet needs (CANE)
								WHELD: 241 (59.65%)	- Mortality
								*Severe*	- **Quality of interactions (QUIS)**
								TAU: 103 (23.23%)	- Pain (APS)
								WHELD: 77 (19.06%)	- Cost
Chenoweth et al. [64]	Australia	Urban residential sites	289	DCM: 83 (7.6) PCC: 84 (6.4) UC: 85 (6.6)	**DCM:** 2 healthcare professionals at each site were trained to become certified mappers in a 2-day course. The remaining staff was trained by the certified mappers and applied PCC plans. Additional support was provided via regular telephone support from experts in DCM. **PCC:** Bradford University training manual was applied in a 2-day training session for staff, central to the practices was a careful review of residents’ life histories.	UC, characterized by custodial and physical task-oriented practices	4 months Follow-up: 4 months	GDS, mean (SD) DCM = 5,6 (1,3) PCC = 5,6 (0,73) CAU = 5,3 (1,1)	*Primary:* - **Agitation (CMAI)**
									*Secondary:*
									- NPS (NPI-NH)
									- QoL (QUALID)
									- **Falls (Records)^c^**
									- Use of antipsychotic drugs (Records)
									- Use of physical restraint (QUIS)
									- Cost of treatment
Chenoweth et al. [63]	Australia	Residential aged care homes	601	CAU = 86 (7) PCC = 84 (8) PCE = 84 (8) PCC + PCE = 84 (7)	**PCC:** Five staff from each of the 19 PCC homes received 32 hours off-site training, which focused on paying attention to the residents’ feelings when agitated, interacting with residents in a person-centered way and using person-centered care planning to meet the residents’ psychosocial needs, followed by on-site supervision in these processes (range 2–16 hours) and telephone support. These staff trained remaining staff after completion of their own training. **PCE:** Included improvements to the safety, accessibility and utility of outdoor spaces, provision of a greater variety of social spaces and using color and objects for way-finding and to improve feelings of familiarity. Two experts in PCE principles planned and supervised implementation of recommended PCE interventions with a maximum budget of AUD$10,000 per home.	UC and UE	4 months, FU: 8 months	GDS severe/very severe in %UC = 88 PCC = 90 PCE = 82 PCC + PCE = 85	*Co-primary outcomes:* - **QoL (DEMQoL self-report and proxy interview)** - **Agitation (CMAI)**^d^ - **Emotional responses in care (ERIC)**^e^ - Depression (CSDD) *Secondary outcome:* - **Care interaction quality (QUIS)**^e^
Cohen-Mansfield et al. [65]	USA	Nursing homes	231	TREA: 85.9 (8.62) Control: 85.3 (9.62) Total: 85.7 (8.89)	**TREA** including individually tailored non-pharmacologic interventions (e.g., simulated social contact, magazine/reading/book on tape (audio drama), music, physical activities, sensory stimulation, puzzles and games, sorting, videos and television, group activities). Delivered by research team (experts in gerontology and psychology).	Placebo intervention (in-service education for care staff members about the syndromes, etiologies, and possible non-pharmacological treatments for agitation).	2 weeks	MMSE Mean (SD) TREA: 7.62 (6.33) Control: 9.38 (6.76) Total = 8.12 (6.48)	*Primary:* - **Agitation (ABMI)** *Secondary:* - **Observed affect (Lawton’s Modified Behavior Stream)**
Eritz et al. [66]	Canada	Nursing homes	73	85.98 (7.49)	**Life history intervention:** Each history, derived from proxy (majorly children and spouses) interviews, was approximately two pages, including one page of photographs, shown to care staff. Family members were encouraged to submit resident’s photographs as well as artefacts from the past to be included. The residents’ life histories or medical histories were written by the primary researcher or a trained research assistant.	Medical history (CAU)	3 months	Average CPS-score (SD): 4.17 (1.57)	- Aggression (ABS) - Agitation (CMAI) - **QoL (ADRQL-R)**
Fossey et al. [67]	UK	Nursing homes	349	Control: 82 (53-101)^*^ Intervention: 82 (60-98)	**PCC-staff training** including an intervention package: care staff were trained in the philosophy and application of PCC. This included ongoing training and group supervision with support and feedback by researchers.	CAU	10 months	CDR, n (%): *None, questionable, or mild* Control: 37/163 (23) Intervention: 25/170 (15) *Moderate* Control: 32/163 (20) Intervention: 46/170 (27) *Severe* Control: 94/163 (58) Intervention: 99/170 (58)	*Primary:* - **Neuroleptic use and dose of neuroleptic** *Secondary:* - Agitation (CMAI) - Quality of life - Proportion of patients taking other psychotropic drugs (Med. records) - Adverse events (including documented falls) (Med. records) - Incidents involving irritable behavior directed at staff or other residents
Lawton et al. [68]	USA	Nursing homes	182	N/A	The **“stimulation-retreat”** model: The intervention program attempted to modulate different perspectives by acknowledging various needs for stimulation both across individuals and at different times within the same person. The major treatment task was to be sensitive to individual preference, individual capability, and contextual appropriateness. The major components of the program were staff training, interdisciplinary care planning, family support, and activity programming, with the choice of a specific type of one-to-one contact being determined by consensus at the care planning session; the most frequent types of contact were conversation, music, reading, or looking at pictures with the resident.	No further information except from “controls”.	12 months	GDS, mean Total (baseline) = 5.53 Total (FU) = 5.87	- Cognitive status (MDRS, GDS) - Functional health (PSMS) - Negative behaviors (BEHAVE-AD) - Agitation (CMAI) - Affective states (incl. depression, externally engaging behaviors) (MOSES) - Externally engaging behaviors (MOSES, Behavior Rating Scale, Activity Participation Scale) - Behavior streams (The Psion event recorder, The Observer, PGCARS,) - Composite factor scores for Problem Behaviors, Depression, Social Quality, and Time Use (MDS)
Rokstad et al. [69]	Norway	Nursing homes	624	Total: 85.7 (8.3) DCM: 85.1 (8.7) VPM: 85.1 (8.5) Control: 87.0 (8.3)	**DCM**: From each participating ward in the intervention group, two care staff attended a DCM course and became certified mappers. The remaining staff were trained in PCC with lectures by the researchers. The certified staff conducted the mapping and trained the remaining staff members. Feedback sessions occurred during the intervention period. **VPM**: From each participating nursing home, two nurses were appointed as VPM coach including the attendance of a VPM-training course. The VPM coaches trained the remaining staff with lectures applying the VPM manual [97].	Placebo incl. DVD with lectures about dementia (no information about PCC) + CAU.	10 months	CDR, mean sum of boxes (SD) Total: 12.8 (4.1) DCM: 12.4 (4.0) VPM: 13.5 (4.4) Control: 12.4 (3.9)	*Primary outcome:* - Agitation (BARS) *Secondary outcomes:* - **NPS (NPI-Q),** - **Depression (CSDD)**^f^ - **QoL (QUALID)**^g^
Sloane et al. [70]	USA	Nursing homes	73	Control: 86.9 (6.1) Intervention: 86.0 (8.6)	**Person-centered showering** sought to individualize the experience for the resident by using a wide variety of techniques, such as providing choices, covering with towels to maintain resident warmth, distracting attention (e.g., by providing food), using bathing products recommended by family and staff, using no-rinse soap, and modifying the shower spray. **The towel bath** is an in-bed method in which the caregiver uses two bath blankets, two bath towels, a no rinse soap, and 2 quarts of warm water; keeps the resident covered at all times; and cleanses the body using gentle massage.	Usual methods of showering	3 months	MMSE, mean (SD): Control: 2.1 (4.1) Intervention: 2.2 (4.0)	*Primary outcomes:* - **Agitation (CAREBA, The Observer Video-Pro)** - **Aggressive behaviors (CAREBA, The Observer Video-Pro)** - **Discomfort (Modified discomfort scale for dementia of the Alzheimer type)** *Secondary measures of effect:* - **Bath duration and completeness (the number of body parts bathed and the number of minutes spent being bathed)** - **Skin condition (Hardy Skin Condition Data Form)** - **Skin microbial flora (Skin Cultures)**
Testad et al. [71]	Norway	Nursing homes	274	- Intervention: 88.2 (8.2) Control: 85.2 (8.2)	The **"Trust Before Restraint"** intervention was based on the evidence of the Relation Related Care (RRC) intervention and decision-making process (DMP), the Norwegian legislation on restraint and best practice for PCC. Included elements of shared decision making and a life history/bibliographical approach.	TAU	7 months	CDR, sum of boxes mean (SD) Intervention: 12.2 (4.8) Control: 12.6 (4.2)	*Primary outcomes:* - **Use of restraint (standardized interview)** *Secondary outcomes:* - **Agitation (CMAI, NPI)** - Use of psychotropic drugs (Medical Journals)
Van Bogaert et al. [72]	Belgium	Nursing homes	72	Total: 84 (78–90)^**^ Intervention: 84 (79.5–90.5) Control: 84 (76–89)	**SolCos** transformational reminiscence model was performed by trained nursing home volunteers as facilitators.	CAU	10 weeks	MMSE: Intervention: 18 (15–22)^**^ Control: 15 (12.5–20)	*Primary outcomes:* - **Depression (CSDD)** *Secondary outcomes:* - Cognition (MMSE, FAB) - Behavior (NPI)
van de Ven et al. [73]	The Netherlands	Nursing homes	268	Intervention: 84.6 (6.1) Control: 83.5 (6.6)	**DCM**: two staff from each care home receiving the intervention were trained and became certified mappers. Initially, an external expert delivered a lecture on PCC. Subsequently, the certified staff conducted the mapping and trained the rest of the staff members. In the beginning of the intervention, members of care staff were given a lecture in both DCM and PCC.	CAU	4 months, FU 8 months	N/A	*Primary outcomes:* - Agitation (CMAI) *Secondary outcomes:* - NPS (NPI-NH) - QoL (Qualidem, EQ-5D)
van der Ploeg et al. [74]	Australia	Residential facilities	57	Total: 78.1 (9.8)	Personalized one-to-one activities that were delivered by a trained psychologist and higher degree psychology student applying **Montessori** principles. Typical selections included listening and singing along to favorite music, looking at and sorting pictures, arranging flowers, sorting dry pastas, folding towels, screwing nuts and bolts together, planting seeds, and making puzzles.	Placebo: social interaction via general conversation	4 weeks	MMSE (range = 0–23) Mean (SD): 6 (8)	*Primary outcomes:* - **Agitation (direct observation and count of frequency of agitated behaviors)** *Secondary outcomes:* - **Affect (PGCARS)** - **Engagement (MPES)**
Van Haitsma et al. [75]	USA	Nursing homes	195	Total: 88.7 (64–105)^****^	**Individualized Positive Psychosocial Intervention (IPPI):** The intervention offered five basic types of activities reflective of the most common resident-preferences. Within each category, two or more specific options were offered (30 activity options total). Physical exercise included the option to take an outdoor walk or work with clay. Music included singing or listening to a favorite artist; reminiscence, reviewing family photos, or writing letters; ADLs, manicures, or preparing a snack; and sensory stimulation could mean a hand massage with lotion or smelling fresh flowers.	UC + attention control	3 weeks	MMSE (range 0–24), mean (SD) Total: 9.0 (7.6)	- Negative affect (sadness, **anger**, anxiety) - **Positive affect (pleasure, alertness)** - **Verbal behavior**^h^ (very negative, negative, positive, **very positive**, no verbal) - Nonverbal behavior (psychosocial task, restlessness, null behavior, eyes closed, aggression, uncooperative, positive touch)^h^ Outcome measures were collected through direct observations in the form of 10-min “behavior streams”, using The Psion event recorder and The Observer software.
van Weert et al. [76]^a^	The Netherlands	Nursing homes	129	Intervention: 84.01 (8.7) Control: 82.60 (8.2)	Staff was trained in principles of **Snoezelen**. The training focused in particular on: the development of CNAs awareness of the residents’ physical, social and emotional needs, making contact with demented residents and showing affection and empathy, supporting demented residents in responsiveness, avoiding to correct the residents’ subjective reality, avoiding to spread useless cognitive information and to test the residents’ remaining cognitive knowledge. The training paid attention to practical skills needed for the application of multi-sensory stimulation, such as taking a life style history interview with family members, arranging a stimulus preference screening to find out which sensory stimuli the resident likes most and writing a snoezel care plan describing how to approach the resident and how to integrate multi-sensory stimuli in 24 h care.	Usual care	18 months	BIP7; 0–21^***^, mean score (SD) Intervention: 14.61 (3.1) Control: 13.37 (4.0)	- **Communicative behavior (RIAS)** - **Nonverbal behavior, e.g., gazing, affective touch, smiling (Observation Scheme with Indicators)**
Boersma et al. [77]	The Netherlands	Nursing homes	212	Intervention: 85.3 (7.5) Control: 85.9 (7.8)	**Veder Contact Method (VCM):** VCM aims to stimulate contact between the person with dementia and the caregiver, by using *theatrical, poetic and musical communication* in combination with elements of existing care methods, that is, reminiscence, validation, and neurolinguistics programming. Care staff were trained in VCM.	CAU	9 months, FU 3 months	MMSE, mean (SD) Intervention: 13.9 (8.9) Control: 14.6 (7.3)	- **QoL (QUALIDEM)** - Behavior and interactions (INTERACT) - Mood (FACE, a three-point Likert scale) DCM to collect observational data on residents and caregivers.
Tay et al. [78]	Singapore	Hospital, Dementia Specific Care Unit	230	Intervention: 82.45 Control: 84.37	**CAMIE:** (1) enhanced medical care protocol, which includes moderating intrusive interventions, a physical restraints-free policy, appropriate and modest use of psychotropic medications, careful attention to hydration, bowel and bladder care, and encouraging mobilization and (2) enhanced psychosocial care protocol, which includes prioritizing patient needs over tasks, encouraging family members and volunteers to provide companionship, and engaging in daily structured activities (e.g., music therapy, recreational/group activities). CAMIE is run by a multidisciplinary team of doctors, nurses, and allied health professionals including a social worker, dietician, pharmacist, as well as physio, occupational, and speech and music therapists.	Conventional geriatric ward	6 months	DSM-IIIR, n and %*Mild* Intervention: 14 (8.20) Control: 2 (3.30) *Moderate* Intervention: 102 (60.00) Control: 37 (61.70) *Severe* Intervention: 54 (31.80) Control: 21 (35.00)	- **Well-being (WB- and IB-Score)** - **Functional ability (MBI)** - **QoL (EQ-5D Index Score)** - **Agitation (PAS)** - Use of psychotropic medications (Medical records) - Length of stay - **Cost-effectiveness**
Verbeek et al. [79]	The Netherlands	Long-term institutional nursing care (i.e., small-scale living facilities and traditional psychogeriatric wards)	259	Intervention: 82.4 (7.9) Control: 83.1 (6.5)	**SSLF**: These facilities were selected based on six characteristics: (1) eight residents per house or unit at most; (2) daily household duties were centered around activities of daily life; e.g., all meals were prepared in the unit’s kitchen by nursing staff together with the residents and/or their family caregivers; (3) staff performed integrated tasks: alongside medical and personal care, they also carried out household chores and organized activities; (4) a small consistent team of staff took care of the residents; (5) daily life was largely determined by the residents, family caregivers, and nursing staff; and (6) the physical environment resembled an archetypal house SSLF are based on a care concept, which emphasizes the normalization of daily life, encourages residents’ participation and autonomy, and a person-centered attitude towards care.	Traditional psychogeriatric wards	12 months incl. FU	MMSE (0-30), mean (SD) Intervention: 11.1 (7) Control: 10.5 (6.6)	*Outcome measures:* - NPS (NPI-NH, CMAI) - Depression (CSDD) *Additional variables:* - **Social engagement (Subscale ISE from RAI-MDS)** - **Use of physical restraint (Questionnaire, type and no. of times)** - **Psychotropic medication (Medical Journals)**
Villar et al. [80]	Spain	Nursing homes	52	Total: 86.7 (7.3)	**ICP program:** Residents were invited to participate in ICP multidisciplinary meetings, attended by staff members (including doctors, nurses, psychologists, social workers and auxiliary CGs) who reached agreements on treatments and recommended intervention strategies. Staff were asked to welcome residents, orientate them in time and space, detail the goals of the meeting, address their interventions to them and take their perspective into account, explain the agreements reached and ask them for their opinion about the treatment and its implementation.	Usual care, i.e., care planning meetings without the patient.	10 months	MMSE, mean (SD): 16.1 (4.0)	- **QoL (GENCAT, proxy-measure)**

Abbreviations: ABMI, agitation behavior mapping instrument, ADRQL-R, Alzheimer’s Disease-related Quality of Life-Revised, APS, Abbey Pain Scale, BARS, Brief Agitation Rating Scale, BEHAVE-AD: Clinical Rating Scale for the Assessment of Pharmacologically Remediable Behavioral Symptomatology in Alzheimer’s Disease, BIP7, Dutch Behavior Observation Scale for Psychogeriatric In-patients Version 7, CANE, Camberwell Assessment of Need for the Elderly, CAU, Care as usual, CAMIE, Care for Acute Mentally Infirm Elders, CAREBA, Care Recipient Behavior Assessment, CDR, clinical dementia rating, CMAI, Cohen-Mansfield’s agitation inventory, CPS, Cognitive Performance Scale, CSDD, Cornell Scale for Depression in Dementia, DCM, Dementia Care Mapping, DemQOL, dementia quality of life, DSM-IIIR, Diagnostic and Statistical Manual of Mental Disorders, DVD, digital video disk, EQ-5D, European Quality of Life 5 Dimensions, ERIC, Emotional Response in Care, FAB, Frontal Assessment Battery, FACE, Face expression scale, FAST, functional assessment staging of Alzheimer’s disease, FU, Follow-up, GDS, Geriatric Depression Scale, GENCAT, Government of Catalonia Scale for Assessment of Residents’ QoL, ICP, Individualized care planning, INTERACT, Mood and Behavior of persons with dementia, ISE, Index of Social Engagement, MBI, Modified Barthel Index, MDRS, Mattis Dementia Rating Scale, MDS, minimum data set, MMSE, mini mental state exam, MOSES, Multidimensional Observation Scale for Elderly Subjects, MPES, Menorah Park Engagement Scale, NPI, Neuropsychiatric Inventory, NPI-NH, Neuropsychiatric Inventory–Nursing Home, NPI-Q, Neuropsychiatric Inventory Questionnaire, NPS, Neuropsychiatric Symptoms, NRS, Non-Randomized Studies, PAS, Pittsburgh Agitation Scale, PCC, Person-Centered Care, PCE, Person-Centered Environment, PGCARS, Philadelphia Geriatric Center Affect Rating Scale, PSMS, Physical Self-maintenance Scale, QoL, Quality of Life, QUALID, quality of life in late-stage dementia, QUALIDEM, Quality of Life of people with Dementia, QUIS, questionnaire for user interaction satisfaction, RAI-MDS, Resident Assessment Instrument –Minimum Data Set, RCT, Randomized Controlled Trial, RIAS, Roter Interaction Analysis System, SD, Standard Deviation, SSLF, Small-scale living facilities, TAU, Treatment as usual, TREA, Treatment Routes for Exploring Agitation, UC, Usual Care, UE, Usual environment, VIPS Framework, valuing people with dementia (V), individualized care (I), understanding the world from the patient’s perspective (P) and providing a social environment that supports the needs of the patient (S), VPM, VIPS Practice Model, WHELD, Improving Wellbeing and Health for People Living with Dementia.

^*^Median (range).

^**^Median (IQR).

^***^The underlined scores indicate the most favorable score (least impairment) for the scale.

^****^Mean (range).

^a^Note: van Weert et al. (2005) applied a quasi-experimental pre- and post-test design, including randomization, hence this study was assessed with RoB2 for risk of bias and is for consistency portrayed in the RCT-category of this table.

^b^Significant effects are marked in bold.

^c^At follow-up, there were fewer falls with DCM than with usual care (*p* = 0.02) and more falls with PCC than with usual care (*p* = 0.03).

^d^Those in PCC + PCE had non-significant changes.

^e^The percentage of positive emotional responses to care (ERIC) improved significantly over time for the PCC + PCE group (by 7%on average, *p* = 0.01), but as the group-by-time interaction was not significant (0.07), differences among groups for emotional responses cannot be inferred. QUIS improvements did not occur in the other groups than PCC + PCE (group-by-time interaction *p* = 0.007).

^f^Significant for VPM.

^g^Significant for DCM. ^h^More negative verbal behaviors by AC- compared to UC or IPPI-groups. AC-group showed more positive behaviors than IPPI; AC- and IPPI-groups showed more positive behaviors than UC-group. The IPPI-group showed significantly more very positive responses than either UC- or AC-groups. Nonverbal responses were significantly higher for the UC-group compared to AC- and IPPI-groups.

### Quality of the included studies

Following the risk of bias assessment with RoB2 [57] for randomized study designs and with NOS [58] for non-randomized study designs, the overall quality of the included studies varied between low to moderate. The results of potential bias assessment in each study are reported in Table 3 for the randomized study designs and Table 4 for the non-randomized study designs.

**Table 3:** Assessment of risk of bias for included RCTs

Author	Randomization process	Deviations from intended interventions	Missing outcome data	Measurement of outcome	Selection of the reported result
Ballard et al. [62]	o	o	o	o	o
Chenoweth et al. [64]	v	o	o	o	o
Chenoweth et al. [63]	o	o	o	o	o
Cohen-Mansfield et al. [65]	o	o	v	v	o
Eritz et al. [66]	v	o	o	v	o
Fossey et al. [67]	o	o	o	o	o
Lawton et al. [68]	o	x	v	v	o
Rokstad et al. [69]	v	o	o	o	o
Sloane et al. [70]	o	o	o	o	o
Testad et al. [71]	v	o	x	o	o
van Bogaert et al. [72]	o	v	v	o	o
van de Ven et al. [73]	o	o	v	v	o
van der Ploeg et al. [74]	o	o	v	o	o
van Haitsma et al. [75]	o	v	o	v	o
van Weert et al. [76]^*^	o	x	v	o	o

Note: Low risk of bias (o), moderate risk of bias (v), high risk of bias (x). Abbreviations: RCTs, randomized controlled trials.

^*^van Weert et al. (2005) applied a quasi-experimental pre- and post-test design including randomization, hence this study was analyzed with Rob2 for risk of bias of included RCTs.

**Table 4:** Assessment of risk of bias for included NRS

Author	Selection	Comparability	Outcome
Boersma et al. [77]^a^	★ ★ ★ ★		★ ★ ★
Tay et al. [78]^b^	★ ★ ★ ★	★ ★	★ ★ ★
Verbeek et al. [79]^a^	★ ★ ★ ★	★ ★	★ ★
Villar et al. [80]^a^	★ ★ ★ ★		★

^a^Prospective, longitudinal quasi-experimental trials, assessed as cohort by proxy, ^b^Prospective naturalistic cohort study. Note: A study can be awarded a maximum of one star for each numbered item within the Selection (4 stars) and Outcome (3 stars) categories. A maximum of two stars can be given for Comparability. Maximum no. of stars in total is nine.

Among the randomized studies, 11 studies [62, 63, 65, 67, 68, 70, 72–76] had a low risk of bias with concern to the randomization process, and four studies had a moderate risk of bias [64, 66, 69, 71]. There was a moderate to high risk of bias for several studies due to deviations from intended interventions [68, 72, 75, 76] or missing outcome data [65, 68, 71–74, 76]. Due to the nature of the study populations, a substantial loss of study participants by decease occurred in the majority of studies, however in three [66, 70, 75] no major loss to follow-up occurred. In general, the authors acknowledged the missing data and reported the reasons. However, none of the studies with moderate to high risk of bias due to missing outcome data [65, 68, 71–74, 76] reported sufficient evidence to judge whether or not their result was biased by missing outcome data, i.e., analysis methods that correct for bias and/or sensitivity analyses. For some studies [65, 66, 68, 73, 75] there were some concerns for risk of bias with regard to the measurement of the outcomes, mostly because blinding of outcome assessors could not be assured. All randomized studies had a low risk of bias in selection of the reported results, i.e., authors were consistent and transparent in the report of their study results.

Among the included NRS, all four studies [77–80] had a low risk of bias associated with the selection process of the exposed and non-exposed cohorts/the experimental and control group. Two studies [78, 79] had a low risk of bias concerning the comparability of cohorts/groups, based on the analysis, while the two other studies [77, 80] had a high risk of bias due to missing information about controlling analyses for confounders and/or covariates. With regards to the outcome assessment (including length and adequacy of follow-up), for two studies [77, 78] there was a low risk of bias, whilst two other studies [79, 80] had some concerns for risk of bias due to self-reported assessments of outcome.

### Synthesis

A summary of key intervention categories, including content (interventions), provider, format, setting, intensity, and fidelity is depicted in Table 5. A total of nine key intervention categories to guide the provision of Person-Centered Dementia Care was identified from synthesis and categorization: 1) social contact, 2) physical activities, 3) cognitive training, including arts/creative activities, 4) sensory enhancement, 5) daily living assistance, 6) life history oriented emotional support, 7) training and support for professional CGs, 8) environmental adjustments, and 9) care organization. The categories including a short description oriented in Dickson et al. [59] and Clarkson et al. [60] are depicted in column one in Table 5.

**Table 5:** Narrative summary of synthesis: intervention categories including descriptions

Intervention category incl. description^*^	Studies (Author(s), year)	Content (Interventions)	Provider^***^	Format	Setting	Intensity	Fidelity^**^
*Social contact:* Provision of different forms of social contact to counterbalance the potentially limited contact with others. This social contact can be real or simulated [60].	Ballard et al. [62], Boersma et al. [77], Cohen-Mansfield et al. [65], Fossey et al. [67], Lawton et al. [68], Tay et al. [78], van der Ploeg et al. [74], van Haitsma et al. [75], Verbeek et al. [79]	Social simulation tool (e.g., robotic animal, lifelike baby doll, baby video, respite video, stuffed animal, family pictures and family video, writing letters) One-on-one interaction (incl. active listening and communication) Conversation (e.g., General and based on e.g., newspaper stories and pictures) Group activity	Trained care staff, researchers in gerontology and psychology, trained psychologist, occupational therapist, nurse, CNAs, rabbi, social workers, a trained multidisciplinary team of doctors, nurses, dietician, pharmacist, physiotherapist, speech therapist, music therapists, volunteers, (higher degree psychology) students, family caregivers	Mostly individual but also and/or group	Nursing home Hospital specialized care unit Residential facilities Long-term institutional nursing care	7AM –3 PM or 3PM –11 PM, 10 min –4 h per week, 1 –7 days per week, 2 weeks –12 months	Substantial loss to follow-up (deaths) yielding high non-completion rates. Lack of staff and time, hence lack of therapeutic communication style in care main obstacles to wider implementation of PCC-interventions. A culture of resistance against intervention / suspicion about intrusion of outsiders among staff and management, hence problem with protocol adherence. Treatment facilitators tempted to deliver intervention to controls when control approach failed. Aggressive or non-cooperative participants. Allocation not randomized, some differences in outcomes existed already at baseline.
*Physical activities:* Provision of structured exercise to create meaningful and engaging experiences that can be a useful counterbalance to difficult behaviors [60].	Ballard et al. [62], Cohen-Mansfield et al. [65], Tay et al. [78], van der Ploeg et al. [74], van Haitsma et al. [75]	Physical activity (e.g., outdoor walks) Gardening	Trained care staff, researchers in gerontology and psychology, a trained multidisciplinary team of doctors, nurses, social worker, dietician, pharmacist, physiotherapist, occupational therapist, speech therapist, music therapists, volunteers, (higher degree psychology) students, CNAs	Individual and/or group	Nursing home Hospital specialized care unit Residential facilities	7AM –3 PM or 3PM –11 PM, 10 min –4 h per week, 1 –7 days per week, 2 weeks –7 months	Substantial loss to follow-up (deaths) yielding high non-completion rates. Lack of staff and time, hence lack of therapeutic communication style in care main obstacles to wider implementation of PCC-interventions. Treatment facilitators tempted to deliver intervention to controls when control approach failed. Aggressive or non-cooperative participants. Problems with protocol adherence.
*Cognitive training:* Provision of stimulation for cognitive functions through a set of standard tasks, which reflect memory, attention or problem solving [60].	Ballard et al. [62], Boersma et al. [77], Cohen-Mansfield et al. [65], Lawton et al. [68], Tay et al. [78], van der Ploeg et al. [74], van Haitsma et al. [75], Verbeek et al. [79]	Puzzles and games Magazine/reading/book on tape Poetry Theatre Arts and crafts (e.g., screwing nuts and bolts together, working with clay, working with fabric) Work like activities, housekeeping tasks (e.g., folding towels) Videos and television Sorting (e.g., sorting pictures, arranging flowers, sorting dry pastas)	Trained care staff, researchers in gerontology and psychology, CNAs, psychologist, rabbi, social workers, a trained multidisciplinary team of doctors, nurses, a social worker, dietician, pharmacist, physiotherapist, occupational therapist, speech therapist, music therapists, volunteers, (higher degree psychology) students, family caregivers	Individual and/or group	Nursing home Hospital specialized care unit Residential facilities Long-term institutional nursing care	7AM –3 PM or 3PM –11 PM, 10 –60 min per week, 1-7 days per week, 3 weeks –12 months	Substantial loss to follow-up (deaths) yielding high non-completion rates. Lack of staff and time, hence lack of therapeutic communication style in care main obstacles to wider implementation of PCC-interventions. A culture of resistance against intervention / suspicion about intrusion of outsiders among staff and management, hence problem with protocol adherence. Treatment facilitators tempted to deliver intervention to controls when control approach failed. Aggressive or non-cooperative participants. Allocation not randomized, some differences in outcomes existed already at baseline.
*Sensory enhancement:* Enhancement or relaxation of the overall level of sensory stimulation in the environment, intended to counterbalance the negative impact of sensory deprivation/stimulation [60].	Ballard et al. [62], Boersma et al. [77], Cohen-Mansfield et al. [65], Lawton et al. [68], Tay et al. [78], van der Ploeg et al. [74], van Haitsma et al. [75], van Weert et al. [76]	Music (e.g., listening, singing along, including in conversations and care) Snoezelen Sensory stimulation (e.g., hand massage with lotion, smelling fresh flowers)	Trained care staff, researchers in gerontology and psychology, CNAs, psychologist, rabbi, social workers, a trained multidisciplinary team of doctors, nurses, a social worker, dietician, pharmacist, physiotherapist, occupational therapist, speech therapist, music therapists, volunteers, (higher degree psychology) students	Mostly individual but also and/or group Individual	Nursing home Hospital specialized care unit Residential facilities	10 min –24 h, 1 –7 days per week, 3 weeks –18 months	Substantial loss to follow-up (deaths) yielding high non-completion rates. Lack of staff and time, hence lack of therapeutic communication style in care main obstacles to wider implementation of PCC-interventions. A culture of resistance against intervention / suspicion about intrusion of outsiders among staff and management, hence problem with protocol adherence. Treatment facilitators tempted to deliver intervention to controls, when control approach failed/intervention was delivered to some control wards. Aggressive or non-cooperative participants.
*Daily living assistance:* Assistance with basic care, e.g., provision of laundry services, basic nutrition and help with activities of daily living [60].	Ballard et al. [62], Cohen-Mansfield et al. [65], Sloane et al. [70], van Haitsma et al. [75], Verbeek et al. [79]	Care (e.g., taking person to bathroom, bringing a sweater or blanket, getting nursing staff, discussing medical condition with physician, repositioning person, taking person to his/her room, bringing eyeglasses, manicure, and other care activities) Food or drink, making snacks Activities of daily living Person-centered showering, towel bath	Trained care staff, researchers in gerontology and psychology, CNAs under supervision of clinical nurse specialist, psychologist or researchers, family caregivers	Individual and/or group	Nursing home Long-term institutional nursing care	7AM –3 PM or 3PM –11 PM, 10 min –4 h per week, 2, 3 or 7 days per week, 2 weeks –12 months	Substantial loss to follow-up (deaths) yielding high non-completion rates. Lack of staff and time, hence lack of therapeutic communication style in care main obstacles to wider implementation of PCC-interventions. Problems with protocol adherence. Allocation not randomized, some differences in outcomes existed already at baseline.
*Life history oriented emotional support:* Support with feelings and emotional needs through discussion or stimulation of memories to enable the person to share their experiences and life stories; intended to counterbalance and help people manage difficult feelings and emotions [60].	Ballard et al. [62], Boersma et al. [77], Chenoweth et al. [64], Eritz et al. [66], Fossey et al. [67], Rokstad et al. [69], Testad et al. [71], van Bogaert et al. [72], van Haitsma et al. [75]	Reminiscence and validation Life history/bibliographical approach interventions	Trained care staff (under supervision of researchers), DCM and VPM champions, special care aides, registered nurses, licensed practical nurses, registered psychiatric nurses, resident care coordinator, trained psychologist, occupational therapist, clinical research nurses, trained nursing home volunteers, supervised CNAs	Individual	Nursing home Urban residential sites	7AM –3 PM or 3PM –11 PM, 10 min –6 h, 2–3 days a week –2 days per 4 months, 2 weeks –10 months	Substantial loss to follow-up (deaths) yielding high non-completion rates. Interruptions in intervention and data collection due to external factors (e.g., influenza outbreak, changes in local laws). Affecting the culture of care within a nursing home. Problems with protocol adherence. Study design did not allow to identify long-term effects nor effect on pharmacological status. Participation decreases in later sessions suggesting necessity to switch over to a maintenance dose.
*Training and support for professional caregivers (CG):* A change of interactions between professional CGs and patients with dementia, including: psycho-education; integrated family support, training in awareness and problem solving; and support groups [59].	Ballard et al. [62], Boersma et al. [77], Chenoweth et al. [64], Chenoweth et al. [63], Eritz et al. [66], Fossey et al. [67], Lawton et al. [68], Rokstad et al. [69], Tay et al. [78], Testad et al. [71], van Bogaert et al. [72], van de Ven et al. [73], van Weert et al. [76], Verbeek et al. [79]	Prof CG education and training (incl. education in antipsychotic drug use) Prof CG support Family support (education/emotional support for family, including family in care decisions)	Trained care staff (under supervision of researchers/external experts from e.g., patient association groups), DCM and VPM champions, special care aides, registered nurses, licensed practical nurses, registered psychiatric nurses, resident care coordinator, trained psychologist, occupational therapist, CNAs, rabbi, social workers, a trained multidisciplinary team of doctors, nurses, a social worker, dietician, pharmacist, physio-, occupational-, speech- and music therapists and volunteers, trained and certified DCM-mappers, family caregivers	Individual and/or group	Nursing home Urban residential sites Residential aged care homes Hospital specialized care unit Long-term institutional nursing care	*Training* 2 –4 days once –4 –7 h twice monthly, 4 –12 months *Supervision:* 2 –16 h once –1 –2 days weekly, 4 –10 months	Substantial loss to follow-up (deaths) yielding high non-completion rates. Inability to control for facility-initiated improvements in the control group. Interruptions in intervention and data collection due to external factors (e.g., influenza outbreak, changes in local laws). Intervention was delivered to some control wards. Problems with protocol adherence/compliance. A culture of resistance against intervention/suspicion about intrusion of outsiders among staff and management, hence problem with protocol adherence. Study design did not allow to identify long-term effects nor effect on pharmacological status. Participation decreases in later sessions suggesting necessity to switch over to a maintenance dose. Allocation not randomized, some differences in outcomes existed already at baseline.
*Environmental adjustments:* Modifications of the living environment, including the visual environment, to ease agitation and/or wandering and promote safety [60].	Ballard et al. [62], Chenoweth et al. [63], Fossey et al. [67], Verbeek et al. [79]	Physical aids, adaptions of environment, assistive technology, signage, reduce noise and clutter, small-scale home-like care environment	Trained care staff, facilitators trained by external experts among staff at each site, trained psychologist, occupational therapist, CNAs, family caregivers	Individual and/or group	Nursing home Residential aged care homes Long-term institutional nursing care	60 min weekly, 1 – 7 days per week, 4 – 12 months	Substantial loss to follow-up (deaths) yielding high non-completion rates. Inability to control for facility-initiated improvements in the control group. Problems with protocol adherence/compliance. A culture of resistance against intervention/suspicion about intrusion of outsiders among staff and management, incl. lack of willingness to make PCE-changes. Allocation not randomized, some differences in outcomes existed already at baseline.
*Care organization:* Connection of different services around the person; advice and negotiation about the delivery of services from multiple providers on behalf of the person [60].	Ballard et al. [62], Chenoweth et al. [64], Chenoweth et al. [63], Fossey et al. [67], Lawton et al. [68], Rokstad et al. [69], Tay et al. [78], Testad et al. [71], van de Ven et al. [73], Verbeek et al. [79], Villar et al. [80]	Interdisciplinary/integrated care planning (incl. consistent staffing), case management Special units (e.g., in hospitals) Shared decision making	Trained care staff (under supervision of researchers), facilitators (e.g., clinical research nurses) trained by external experts among staff at each site, DCM and VPM champions, trained psychologist, occupational therapist, CNAs, rabbi, social workers, a trained multidisciplinary team of doctors, nurses, a social worker, dietician, pharmacist, physio-, occupational-, speech- and music therapists and volunteers, trained and certified DCM-mappers, family caregivers	Individual and/or group	Nursing home Urban residential sites Residential aged care homes Hospital specialized care unit Long-term institutional nursing care	20 min –6 h, 2 days per week, 2 weeks –12 months	Substantial loss to follow-up (deaths) yielding high non-completion rates. Inability to control for facility-initiated improvements in the control group. Problems with protocol adherence/compliance. A culture of resistance against intervention/suspicion about intrusion of outsiders among staff and management, incl. lack of willingness to make PCE-changes. Interruptions in intervention and data collection due to external factors (e.g., changes in local laws). Allocation not randomized, some differences in outcomes existed already at baseline.

Abbreviations: CNAs, Certified Nurse Aides; DCM, Dementia Care Mapping; VIPS Framework, valuing people with dementia (V), individualized care (I), understanding the world from the patient’s perspective (P) and providing a social environment that supports the needs of the patient (S); VPM, VIPS Practice Model.

^*^Oriented in Dickson et al. [59] and Clarkson et al. [60].

^**^As indicated in text, where concrete information about the interventions’ implementation process could not be identified, we report information about problems and/or (methodological) limitations the authors faced.

^***^As the multi-component intervention studies included several interventions, which allowed for categorization of the study in several categories, some listed provider descriptions are repeated in several columns.

#### Content

The PC-interventions followed heterogeneous approaches under the concept of PCC and details available with regard to the description of the delivered PC-interventions, i.e., what was delivered to the PlwD, varied, especially for the multi-component interventions [62–65, 67–69, 73, 74, 77–79]. Some (e.g.,[65]) provided detailed lists of activities included in their multi-component interventions. Others more generally described the provided multi-component interventions as “PCC”, without detailed information about the concrete activities provided to the patients [67, 69] or scarcely described information about activities included [64]. Multi-component interventions with detailed descriptions about each intervention component were respectively assigned to several categories. Some studies limited their intervention-descriptions to the trainings provided to the professional CGs, but did not provide details about which interventions were delivered to the PlwD [64, 67, 69]. Interventional studies conducted under the term RCC aimed at an effect among the PlwD that fit eligibility criteria could not be identified.

#### Provider

Details about the provider(s) were generally described well throughout all included studies. Interventions were delivered by a range of professional CGs, researchers, volunteers, and family CGs. Professional CGs usually received a specified training, some studies had a particular focus on CG training and support, e.g.,education in antipsychotic drug use and regular supervision by researchers or external experts in PCC [62–64, 66–69, 71–73, 76–79]. Some multi-component interventions incorporated, aside from intervention components for the PlwD, education and support for family CGs or otherwise inclusion of the family CGs in care decisions [62, 67, 68, 78, 79].

#### Format and setting

The format differed according to the respective intervention category, but both individual and group formats were applied. The predominant setting was long-term institutional care, except from one study which was conducted in a hospital [78].

#### Intensity

There was a substantial variation in the intensity of the delivered interventions and detailed information was not available in all studies. Some studies chose a short overall timeframe of a few weeks [65, 74, 75], others up to 18 months [76]. Table 5 captures the ranges (min. and max.) of different timeframes applied in the studies for each distinctive intervention category, i.e., time of the day, how many minutes/hours per week, how many days per week, how many weeks per month and so on.

#### Fidelity

Where the included studies contained little information on the delivery process of the interventions, it was challenging to judge their fidelity, i.e., had the intervention always been delivered as intended or had there been challenges to delivery [61]. The term “fidelity” was only mentioned in two studies, [77] and [75]. Where concrete information about the interventions’ delivery process could not be identified, information about problems and/or (methodological) limitations is reported. All studies of longer duration faced problems with a loss to follow-up, due to participants’ decease, which resulted in high non-completion rates. Some reported failure to show a significant effect may reflect difficulties inherent in affecting the culture of care within a nursing home [67], including resistance against the intervention and suspicion about the intrusion of outsiders (i.e., the researchers) among care staff and the management [63, 68]. Some studies reported problems with protocol-adherence [72–75], including provision of the intervention in the control groups [76]. In some studies [66, 71], external factors (e.g.,influenza outbreaks on sites, changes in national laws to restrict use of restraint) were discussed to have influenced the outcome of the intervention.

## DISCUSSION

This systematic review identified a total of nine key intervention categories to guide the provision of person-centered dementia care. The categories comprised a wide range PC-interventions, oftentimes delivered as multi-component interventions, which followed heterogeneous approaches under the concept of PCC. Details in description of the interventions, especially the multi-component interventions, varied. Interventional studies conducted under the term RCC aimed at an effect among the PlwD that fit eligibility criteria could not be identified. The predominant setting was long-term institutional care. No studies were undertaken with PlwD at home. The overall quality of the included interventional studies varied between low to moderate.

The key intervention categories were oriented in those named by earlier reviews [59, 60]. However, Clarkson et al. [60] performed a review of systematic reviews about psychosocial interventions, without a particular emphasis on PCC and interventions published under this concept. In our categorization of PC-interventions, “arts/creative activities” were not allocated their own category, even though they constitute an important segment of PC-activities. However, “music” or “to make music” made this allocation challenging, as some may recognize this as part of arts/creative activities in line with Schneider [81], while others may recognize this as “sensory enhancement” in line with Dickson et al. [59] and Clarkson et al. [60]. Respectively to previous research, arts/creative activities were categorized under cognitive training [82, 83] and music under sensory enhancement [59, 60]. The in this study identified and categorized PC-interventions were similar to the psychosocial interventions identified by Dickson et al. [59] and Clarkson et al. [60]. Future research may want to consider a clearer differentiation between psychosocial interventions and PC-interventions. It may be that PCC is a subset of psychosocial interventions, or the opposite, as PCC by some arguably could be conceptualized in clinical interventions as well, cf. ‘personalized medicine’.

The variation in descriptions of the PC-interventions, especially the multi-component interventions, made the judgement and decision about categorization, as well as descriptions of content, provider, format, intensity, and fidelity, challenging. Only a few concretely reported an assessment of preferences and or needs prior to the intervention, among which only three assessed preferences by a direct involvement of the PlwD. Additionally, no study with multi-component interventions provided a detailed description of which exact activity was delivered to whom, by whom, for how long, and aimed at which outcome measure. Thus, it cannot be differentiated which single activity from the multi-component interventions yielded a potentially significant effect. Generally, it may be considered, whether effectiveness of PC-interventions can be determined in a study, where the intervention was implemented for two weeks [65]. However, a recent systematic review and meta-analysis by Kim and Park [47] identified a significant effect to reduce agitation for the two-week-intervention by Cohen-Mansfield et al. [65]. Aside from agitation, Kim and Park [47] found PC-interventions to reduce neuropsychiatric symptoms, and depression, as well as to improve the quality of life. Their review included some of the studies as we included in our review [63–65, 67, 69, 73, 74]. Similar to our review, Kim and Park [47] did not distinguish between multi-component interventions and single-component interventions for their assessment of the effectiveness of PC-interventions. Future work with PC-interventions may want to consider a clearer differentiation between multi-component interventions and single-component interventions, to increase the accuracy in assessment of PC-interventions for key intervention categories, including potential assessments of relative effectiveness. Additionally, future research may want to consider a standardization for the report of PC-interventions in studies and respective research papers. This includes more detailed descriptions on what it is that constitutes “person-centered” in this intervention, such as preferences-/needs-assessments and/or relationship facilitation and here upon provided interventions, to increase comparability and identify a common approach under the concept of PCC. The definition of an appropriate time frame for the provision of PC-interventions to measure their effectiveness might be valuable.

Despite the inclusion of RCC in the search string to account for the aforementioned development of the PCC concept, we could not identify interventional studies conducted under this concept that met our eligibility criteria. The importance of relationships was built into Tom Kitwood’s original formulations, although in PCC concepts built upon the relational aspect are invariant [19, 28]. Current experiences during the ongoing COVID-19 pandemic underline the need to focus more on the relationship between PlwD, their significant others, and providers [84, 85]. Furthermore, it may be interesting to analyze how COVID-19 affects the capacity of care organizations to deliver person-centered dementia care. It may be interesting for future studies to evaluate the relative effectiveness of “person-centered”- including “relationship-centered”-interventions to assess whether an explicit focus on relationships around PCC-interventions yields an added benefit, not just for the receivers of care but also for the providers. A review of lay literature on PCC for PlwD may be valuable.

The predominant setting was long-term institutional care, which is similar to findings by Kim and Park [47]. The operational model (for profit or not for profit) of the long-term care facilities in the included studies could not be identified. Future research may want to examine whether respective institutions have tendencies to implement certain types of PC-interventions. Aside from the operational model of long-term care facilities, an examination of whether a potential culture change movement in long-term care promotes PCC for PlwD would be interesting. Only a few reported on cultural change in the intervention facilities [63, 67, 68, 77], however, with rather negative observations. Future research on PCC in long-term institutional care facilities may want to examine, whether a potential cultural change that promotes the provision of PCC for PlwD nevertheless is underway, e.g.,by a review of qualitative research with both professional and family CGs.

No studies were undertaken with PlwD at home. It is recognized that the concept of PCC has been developed and implemented with a focus on residential homes for the aged [38, 47]. The choice of setting could also be associated with the human and financial resources required to deliver PC-interventions to PlwD at home. Additionally, PC-interventions for PlwD at home might not have been identified by the term “intervention”. For this reason, “home services” instead of “intervention” as third dimension was included during pilot searches, which, however, yielded a scarce number of hits. Kim and Park [47] identified two studies conducted in people’s homes [86, 87], both of which applied the term “intervention”. We did not find a study conducted with PlwD at home and only one PC-intervention study at a hospital. Two recent systematic reviews [88, 89] focused their research on needs of PlwD and registered nurses’ experiences with PCC in the hospital setting. As there is an aim by policy makers to move care delivery to the home [90] and many aged people prefer to receive care at home [91], this setting should find greater consideration in future investigations about PCC and PC-interventions. Aside from the home/primary care setting, future research may want to consider a greater focus on hospital settings with particular focus on assessment of patients’ needs and training for staff.

The overall quality of the included interventional studies varied between low to moderate, similar to findings by Kim and Park [47], who remarked future research should focus on utilization of precise methods for randomization, allocation concealment, and blinding of those who collect the data, to confirm validity of findings in systematic reviews. In this review, most studies had a low risk of bias with regard to the randomization process. However, assessment blinding likewise formed ground for risk of bias in many studies, as did a substantial loss to follow up due to participants’ decease in most studies. Still, the nature of the included populations, i.e., people of very high age, as well as the type of interventions assessed, i.e., psychosocial non-pharmacological interventions, which are known to pose a challenge with regards to blinding of assessors, should be remembered. Hence, in line with previous literature [47], more studies with rigorous designs are recommended to address the aforementioned areas for future research with an evidence base of sufficient high-quality.

### Limitations

This review has several limitations. Despite great efforts, including an extensive review of the MeSH-database [51] and previous literature, e.g.,[38, 52, 53] to develop a comprehensive list of terms for PCC and a thoroughly piloted search, we cannot be fully certain to have identified all terms that comprise all PC-interventions for PlwD. PC-interventions for PlwD at home might be covered by the term “community care”, as suggested in [27, 92, 93], which was not included in the search string. However, we included terms such as client-centered, consumer-centered, client-focused, person-focused, client-directed, and consumer-driven care, noted by [38, 52, 53], to identify PCC and PC-interventions for PlwD at home. Furthermore, eligible interventional studies conducted under the concept of RCC might have been covered by terms focused on “family involvement” [94], which was not included in the search string. Future reviews should pay particular attention to the choice of terms to identify interventions conducted under the RCC-concept and in the home care setting, i.e., to apply a broad lens during the development of the search string. Similar to Kim and Park [47], our small sample size of papers that fit into the defined parameters limits the effectiveness to capture the varied interventions that may be available under the concept of PCC. It could be that important ideas and interventions were discarded due to the qualitative nature of research needed to capture the effectiveness of interventions attempted in real life care situations without controlled settings, which is a major limitation of this review focused on published PC-interventional research. Hence, a further review with less strict inclusion parameters including published lay literature might be of value to capture PCC-initiatives outside the published academic literature. Searches could have been performed in further databases to raise sensitivity, however, with the chosen combination of databases, we hope to have identified all relevant records and inclusion of additional databases was not expected to yield additional information. Due to language skills in the team, we only included English and German records, which might have excluded other eventually relevant studies. Even though we applied a thorough protocol and strategy for study selection, data extraction, risk of bias assessment, and synthesis, we cannot rule out potential errors in any of the systematic steps. However, since every step of this systematic review entailed a review by several reviewers, these potential errors were minimized. This study applied the NOS Cohort risk of bias tool non-adapted for the included quasi-experimental studies, which is not ideal. Nevertheless, as the particular studies had prospective and longitudinal designs, we considered this approach acceptable in terms of pragmatism, simplicity in use and due to lack of a better, equally validated tool. No statistician was involved in the risk of bias assessment. However, several reviewers in the team (AR, BM, MR, WH) hold senior level experience with statistical methods, and guided the two main reviewers (WM and AA). The heterogeneity in reporting and application of the PCC-concept in the included interventional studies makes comparisons both within this review and with other reviews such as Kim and Park’s [47] difficult. Still, our detailed approach to identify key intervention categories for better guidance on the provision of person-centered dementia care, including who did what, where, and how, is an attempt to provide an opportunity for better comparison of PC-interventions. Protocols and process-evaluations of the included studies were not checked, as these would not comply with eligibility criteria and per study, only one published record was included. Any deviations from protocol were expected to be mentioned in the published reports on findings. Judgements about the dementia severity and the inclusion criterion, whether a diagnosis by health professionals exists, were challenging as this was rarely reported. Some had a dementia diagnosis as inclusion criterion and reported this [63, 64]. However, with the exception of one study [73], all remaining studies reported on assessed dementia scores with validated tools (see 2nd column from the right in Table 2), which indicated dementia severity. Van de Ven et al. [73] conducted their study at Dementia Special Care Units. Hence, we interpreted the eligibility criterion with regard to dementia severity criterion to be fulfilled. We did not perform a meta-analysis, as an assessment of relative effectiveness only recently has been reported [47]. The quality of a meta-analysis with a wide range of various outcome measures, as included in this review, would have been questionable. Furthermore, this review analyzed the distinct activities performed under the scheme of the PC-interventions, as a result of which the multi-component interventions are listed in several categories. For an assessment of relative effectiveness, the interventions need to be assessed as a whole, cf. [47], which contradicts the strategy of this review. Finally, it may seem at odds with the notion of PCC as a holistic philosophy of care, to refer to discrete interventions and intervention categories of person-centered dementia care. In this regard it may further be questioned, whether PCC is just good care, as suggested by some [6], and accordingly be recognized that good care manifests in different ways in different contexts and hence probably is hard to categorize and standardize. Nevertheless, to offer clearer guidance on the provision of person-centered dementia care, including who does what, where and how, information about key intervention categories of person-centered dementia care needed to be identified, as this review provides the evidence for.

## CONCLUSIONS

This systematic review provides a current state overview of published PC-interventional studies in dementia and identified nine key categories to provide person-centered dementia care, including who did what, where and how. the interventions followed heterogeneous approaches under the concept of person-centered dementia care. this heterogeneity made it challenging to identify a similar approach of person-centered dementia care and respective key intervention categories. Future research may want to consider a clearer differentiation between multi-component- and single-component interventions to operationalize the theoretical person-centered dementia care concept under a homogenous approach. Furthermore, attention to an appropriate time frame for the provision of PC-interventions with regard to effectiveness assessments may be considered.

## Supplementary Material

Supplementary Material
